# Functional brain controllability in Parkinson’s disease and its association with motor outcomes after deep brain stimulation

**DOI:** 10.3389/fnins.2024.1433577

**Published:** 2024-11-07

**Authors:** Ziyu Li, Zhiqin Liu, Yuan Gao, Biqiu Tang, Shi Gu, Chunyan Luo, Su Lui

**Affiliations:** ^1^Huaxi MR Research Center (HMRRC), Functional and Molecular Imaging Key Laboratory of Sichuan Province, Department of Radiology, West China Hospital of Sichuan University, Guoxue Xiang, Chengdu, China; ^2^Research Unit of Psychoradiology, Chinese Academy of Medical Sciences, Guoxue Xiang, Chengdu, China; ^3^Department of Neurosurgery, West China Hospital of Sichuan University, Chengdu, China; ^4^School of Computer Science and Engineering, University of Electronic Science and Technology of China, Chengdu, China

**Keywords:** Parkinson’s disease, deep brain stimulation, functional controllability, motor outcomes, cortico-striato-thalamic-cortical (CSTC) motor loops

## Abstract

**Introduction:**

Considering the high economic burden and risks of deep brain stimulation (DBS) surgical failure, predicting the motor outcomes of DBS in Parkinson’s disease (PD) is of significant importance in clinical decision-making. Functional controllability provides a rationale for combining the abnormal connections of the cortico-striato-thalamic-cortical (CSTC) motor loops and dynamic changes after medication in DBS outcome prediction.

**Methods:**

In this study, we analyzed the association between preoperative delta functional controllability after medication within CSTC loops and motor outcomes of subthalamic nucleus DBS (STN-DBS) and globus pallidus interna DBS (GPi-DBS) and predicted motor outcomes in a Support Vector Regression (SVR) model using the delta controllability of focal regions.

**Results:**

While the STN-DBS motor outcomes were associated with the delta functional controllability of the thalamus, the GPi-DBS motor outcomes were related to the delta functional controllability of the caudate nucleus and postcentral gyrus. In the SVR model, the predicted and actual motor outcomes were positively correlated, with *p* = 0.020 and *R* = 0.514 in the STN-DBS group, and *p* = 0.011 and *R* = 0.705 in the GPi- DBS group.

**Discussion:**

Our findings indicate that different focal regions within the CSTC motor loops are involved in STN-DBS and GPi-DBS and support the feasibility of functional controllability in predicting DBS motor outcomes for PD in clinical decision-making.

## 1 Introduction

Deep brain stimulation (DBS) is an effective therapy for Parkinson’s disease (PD) and plays a vital role in improving motor function and quality of life in patients experiencing motor fluctuations and dyskinesia uncontrollable by optimal medical therapy (Perlmutter and Mink, 2006; Ramirez-Zamora and Ostrem, 2018; Hayes et al., 2019; Armstrong and Okun, 2020). The two main targets of DBS treatment in PD are the sub-thalamic nucleus (STN) and globus pallidus interna (GPi). As an invasive and expensive treatment, appropriate candidate selection before DBS implantation is crucial to reduce the disease and economic burden on patients with PD (Becerra et al., 2016; Fann et al., 2020). Despite the general selection criteria, including excellent levodopa response, younger age, no or very mild cognitive impairment, and the absence of well-controlled psychiatric disease (Hariz and Blomstedt, 2022; Pollak, 2013), inter-individual heterogeneity of DBS motor outcomes has been observed in several studies (Younce et al., 2019; Kluger et al., 2011). Therefore, identifying more pre-surgical characteristics associated with DBS motor outcomes would be of significant assistance in clinical decision-making.

The preoperative levodopa challenge test (LDCT) is widely accepted for outcome prediction and candidate selection for DBS (Pollak, 2013). However, some studies have suggested that preoperative levodopa responsiveness and DBS benefits are not congruent (Piboolnurak et al., 2007; Zaidel et al., 2010) and that LDCT alone cannot predict DBS outcomes (Lin et al., 2021). Chen et al. reported that the preoperative response to levodopa had a generally good predictive value in the improvement of rigidity and bradykinesia; however, poor performance in total motor symptoms (Chen et al., 2022). Moreover, some patients with suboptimal preoperative LDCT results could still benefit from DBS implantation (Morishita et al., 2011; Yamada et al., 2008). Because LDCT alone is far from sufficient for DBS outcome prediction, new quantitative preoperative indicators are required.

Brain network dysfunction is a crucial pathophysiological progression in PD, mainly involving the sensorimotor networks (Baggio et al., 2015; Tessitore et al., 2019; Ruppert et al., 2020; De Micco et al., 2021). Compared to that in non-candidates, altered functional connectivity of the sensorimotor networks was observed in DBS candidates (Albano et al., 2022), and was correlated with the clinical outcomes of PD patients (Horn et al., 2017). Although the therapeutic effects on motor symptoms of DBS were possibly modulated by networks involving stimulation targets and other motor-related areas of the brain, generally recognized as cortico-striato-thalamic-cortical (CSTC) motor loops (Lozano and Lipsman, 2013), it remains unclear how the therapeutic effects of subthalamic nucleus DBS (STN-DBS) and globus pallidus interna DBS (GPi-DBS) are modulated by specific regions within the CSTC motor loops. In addition, to our knowledge, levodopa response to brain dynamics has rarely been considered in previous studies when predicting the DBS motor outcomes.

Network control theory provides a rationale for exploring the ability of a particular node to drive the whole brain system into specific states (Gu et al., 2015), and can be used to predict whether the effects of stimulation remain focal or spread globally (Muldoon et al., 2016). In other words, network controllability can be used to evaluate the contributions of different nodes to an entire motor circuit or network. Additionally, functional controllability can be used to evaluate connectivity and dynamic brain changes over time (Li et al., 2023). The average controllability (AC) and modal controllability (MC) are the two most commonly used network control indicators. Regions with a high AC can drive the brain to easily reach states, whereas regions with a high MC can drive the brain to states that are difficult to reach. Abnormal regional network controllability has been reported in several neuropsychiatric diseases. AC in the transmodal cortex can help predict positive psychotic spectrum symptoms (Parkes et al., 2021). Furthermore, Zarkali et al. (2020) found that reduced AC correlated with the presence of hallucinations in Parkinson’s disease. However, the relationship between network controllability and the motor outcomes of DBS in patients with PD has not been fully elucidated.

In this study, we evaluated the AC and MC of each brain region of PD patients at medication “on” and medication “off” states before DBS surgery, and the change of controllability between “on” and “off” states (here we termed delta controllability). We then assessed the association between the delta controllability of the focal regions and motor outcomes of STN-DBS and GPi-DBS in patients with PD, and the feasibility of delta controllability in predicting DBS motor outcomes. We hypothesized that: (1) compared to healthy controls, controllability of PD patients would change significantly in focal regions within CSTC loops involving the striatum and motor-related cortices, which could be reversed by medication; and (2) preoperative delta controllability of those regions could be related to the motor outcomes of STN-DBS and GPi-DBS, with different involvement of regions in CSTC loops between both groups.

## 2 Methods

### 2.1 Participants

Fifty participants who were diagnosed with idiopathic PD according to the UK Brain Bank criteria were recruited according to the CAPSIT-PD recommendations (Defer et al., 1999; Umemura, 2023) as movement disorder outpatients at the West China Hospital of Sichuan University (Chengdu, China). All patients showed good responses to levodopa with more than 30% improvement in the motor performance. Among the 50 patients with PD prospectively included in this study, five patients without surgery, two patients who had unilateral DBS implantation, and one patient with radio-frequency lesions were excluded. Three patients were excluded due to missing images or incomplete clinical data. The final sample included 39 patients, all of whom underwent bilateral STN-DBS or GPi-DBS in accordance with standard clinical procedures at the Neurosurgery Department of the West China Hospital of Sichuan University (Chengdu, China). Additionally, 29 healthy controls (HCs) with no history of neurological or psychiatric diseases were recruited through community advertisements in Chengdu, China.

### 2.2 Clinical evaluation

Before DBS surgery, a detailed medical history review and neurological examination were performed to exclude dementia or other neuropsychiatric disorders. The assessments of motor symptoms included two time points: preoperative and 1 month postoperatively when all cases began electrical stimulation. Motor symptoms were evaluated using the third part of the Unified Parkinson’s Disease Rating Scale (UPDRS-III) after withdrawal of dopaminergic medication for at least 12 h. To evaluate the therapeutic effects of DBS on motor function, the UPDRS-III score was rated after surgery under DBS stimulation without medication (medication-off/stimulation-on state). To evaluate the overall motor outcomes of DBS, we used the following formula: improvement rate = [(preoperative UPDRS-III score on medication-off state–postoperative UPDRS-III score on medication-off/stimulation-on state)/preoperative UPDRS-III score on medication-off state] × 100%.

### 2.3 Data acquisition and pre-processing

Resting-state functional MRI images were acquired for each HC and each patient before and after dopamine replacement medication, using 750 W 3.0 T (General Electric, Milwaukee, WI) scanners with the following sequence: TR = 2000 ms, TE = 35 ms, flip angle = 90°, acquisition matrix = 64 × 64, field of view (FOV) = 23 cm × 23 cm, slice thickness = 3.6 mm, 33 slices, and a total of 235 time points.

Before preprocessing, we examined all the images to guarantee their quality and excluded data with apparent artifacts or brain abnormalities. Resting-state functional MRI scans were preprocessed using Statistical Parametric Mapping (SPM12)^1^ and Resting-State fMRI Data Analysis Toolkit Plus (RESTplus, Version 1.24; Jia et al., 2019) based on the MATLAB 2017b platform. For each participant, all images were converted from DICOM format to NIFTI format. The first 12 time points were discarded, leaving 223 time points for the following steps including slice timing correction, head motion correction, and spatial normalization to the Montreal Neurological Institute (MNI) template. We checked the images and head motion parameters after normalization and removed scans with head motion of more than 3 mm or those that failed to normalize. Two scans at the medication-off state and 5 scans at the medication-on state were excluded due to the inevitable head motion in patients with advanced Parkinson’s disease. Then, a 6 mm FWHM Gaussian kernel was used to smooth the images and reduce spatial noise. Subsequently, the global mean signal, 24 head motion parameters, white matter signal, and cerebrospinal fluid signal were regressed to reduce the effect of head motion on functional connectivity. Finally, a band-pass filter (0.01–0.08 Hz) was used to remove low-or high-frequency noise and artifacts. FC metrics were constructed from 90 regions of interest (ROIs) parcellated using an automated anatomical labeling (AAL) atlas (Tzourio-Mazoyer et al., 2002). Head motion may systematically alter correlations in functional connectivity (FC). We also conducted head motion scrubbing to further reduce the influence of the head motion and to strengthen the reliability of our analysis as recommended (Ciric et al., 2017; Power et al., 2014), the results were similar to those without head-motion scrubbing (details are presented in the Supplementary materials).

### 2.4 Functional network controllability

Network controllability evaluates the ability of a single brain area to drive the entire brain from one state to another with an external energy input. We calculated the AC and MC from functional connectivity of each HC and each patient at both medication “on” and “off” states, using packages and codes from Gu’s research (Gu et al., 2015), available.^2^

As mentioned in previous studies on network controllability (Gu et al., 2015; Li et al., 2023; Tang et al., 2023), the dynamics of neural activity were estimated using a simplified noise-free linear discrete-time and time-invariant model, mathematically described as


$$x\left(t+1\right)=\mathrm{Ax}\left(t\right)+B_{K}u_{K}\left(t\right)\text{,}$$

Where $x\in R^{N}\;\left(N=90\right)$ , describes the brain states of BOLD activity at a given time t , and $A\in R^{N\times N}$ is the weighted and symmetric adjacency matrix. We calculated matrix A from functional connectivity because studies have found that functional controllability may better reflect real brain dynamics (Scheid et al., 2021) and allow for dependence on different states (Deng et al., 2022) than structural controllability. $B_{K}$ is the input matrix, and 90 ROIs from the AAL atlas were set as control points K in our study. The $u_{K}\left(t\right)$ is the energy applied to the control point to drive the state transition and is proportional to the inverse of the controllability Gramian matrix.

AC and MC were most commonly used in previous studies. AC is equivalent to the trace of the controllability Gramian matrix:


$$W_{K}=\overset{\infty }{\underset{\tau =0}{\sum }}A^{\tau }B_{K}B_{K}^{T}A^{\tau }\text{.}$$

Here, τ indicates the time step of the trajectory, which is set to infinity. Because $W_{K}$ is inversely proportional to the control energy $u_{K}\left(t\right)$ required for brain state shifting, brain regions with higher AC can drive the brain into easy-to-reach states with lower energy costs.

The MC of brain region i is calculated from the eigenvector matrix $v_{ij}$ of matrix A which is mathematically defined as


$$\emptyset_{i}=\overset{N}{\underset{j=1}{\sum }}\left(1-\lambda_{j}^{2}\left(A\right)\right)v_{ij}^{2}\text{.}$$

Regions with high MC can more easily drive the dynamics of a brain network toward hard-to-reach states for goal-specific operations with high energy costs.

The change of controllability between medication “on” and “off” states of patients with PD, termed as delta controllability, were also calculated to explore the effects of dopamine replacement medication on brain dynamics.

### 2.5 Statistical analysis

Statistical analyses were performed using the Statistical Package for the Social Sciences (SPSS, version 24) and functions from the R statistics toolbox. We employed chi-square tests and t-tests to measure differences in the demographic and clinical characteristics between patients with PD and HCs and between the STN-DBS and GPi-DBS groups.

Covariance analyses were used to compare differences in AC and MC between patients with PD and HCs. Paired *t*-tests were used to measure the changes in the AC and MC before and after medication in patients with PD. We counted brain regions that not only had significant differences between patients and HCs but also changed significantly after medication.

Given that the basal ganglia and cortical sensorimotor areas are critical regions of PD pathology, Pearson’s correlations between delta controllability of these brain regions (caudate nucleus, putamen, globus pallidum, thalamus, precentral gyrus, postcentral gyrus, supplementary motor area, and paracentral lobule) and percentage change in UPDRS-III after DBS surgery were conducted in the STN and GPi groups.

We then constructed support vector regression (SVR) models based on the LIBSVM toolbox with leave-one-subject-out cross-validation (LOOCV) to explore whether delta controllability in the regions found in the previous procedures (Table 1) could predict the improvement rates of UPDRS-III after DBS surgery in the STN-DBS and GPi-DBS groups.

**Table 1:** Correlation between delta controllability and improvement rates of the UPDRS-III scores.

Location	Type of controllability	Brain region^a^	Hemisphere	Pearson’s correlation	
				R	*p*
STN	Average	THA	Left	−0.687	0.001
STN	Average	THA	Right	−0.624	0.003
GPi	Average	PoCG	Left	0.744	0.006
GPi	Average	PoCG	Right	0.643	0.024
GPi	Modal	PoCG	Left	−0.584	0.046
GPi	Modal	CAU	Left	0.592	0.042

a THA, Thalamus; PoCG, Postcentral gyrus; CAU, Caudate nucleus.

## 3 Results

### 3.1 Participants and clinical evaluation

The demographic information of the patients with PD and HCs, together with the manifestations of PD, are summarized in Table 2. Thirty-nine patients and 29 healthy controls were included in the final sample. Among the 39 patients with PD included in this study, 24 underwent STN-DBS DBS and 15 underwent GPi DBS. Age, sex, motor subtypes, duration, age at disease onset, and UPDRS-III scores in the medication-off and medication-off/stimulation-on states showed no significant differences between the STN-DBS and GPi-DBS groups.

**Table 2:** Demographic characteristics of participants.

Attributes	Patients *N* = 39	HCs *N* = 29	T or χ^2^	*p* value	STN group *N* = 24	Gpi group *N* = 15	T or χ^2^	*p* value
Age (years)	59.21 ± 9.61	53.48 ± 8.40	2.560	0.013	61.79 ± 8.82	56.67 ± 10.57	1.316	0.196
Sex (Male/Female)	21/18	8/21	4.689	0.030	12/12	9/6	0.371	0.542
Subtypes (TD/PIGD/I)^a^	10/16/13	–	–	–	9/7/8	1/9/5	5.562	0.062
Duration (years)	8.44 ± 3.35	–	–	–	7.96 ± 3.47	9.20 ± 3.12	1.129	0.266
Onset age (years)	50.77 ± 10.32	–	–	–	52.83 ± 10.04	47.47 ± 10.21	1.613	0.115
Hoehn & Yahr	3.32 ± 0.81	–	–	–	3.21 ± 0.78	3.50 ± 0.85	1.101	0.278
UPDRS-III at medication-off state before surgery	55.95 ± 16.37	–	–	–	56.08 ± 15.83	55.73 ± 17.76	0.064	0.946
UPDRS-III at medication-off/stimulation-on state	17.54 ± 10.29	–	–	–	16.96 ± 11.00	18.47 ± 9.32	0.441	0.662

a TD, tremor-dominant; PIGD, postural instability and gait difficulty-dominant; I, intermediate.

### 3.2 Aberrant brain regions of controllability responsive to medication

**Figure 1:**
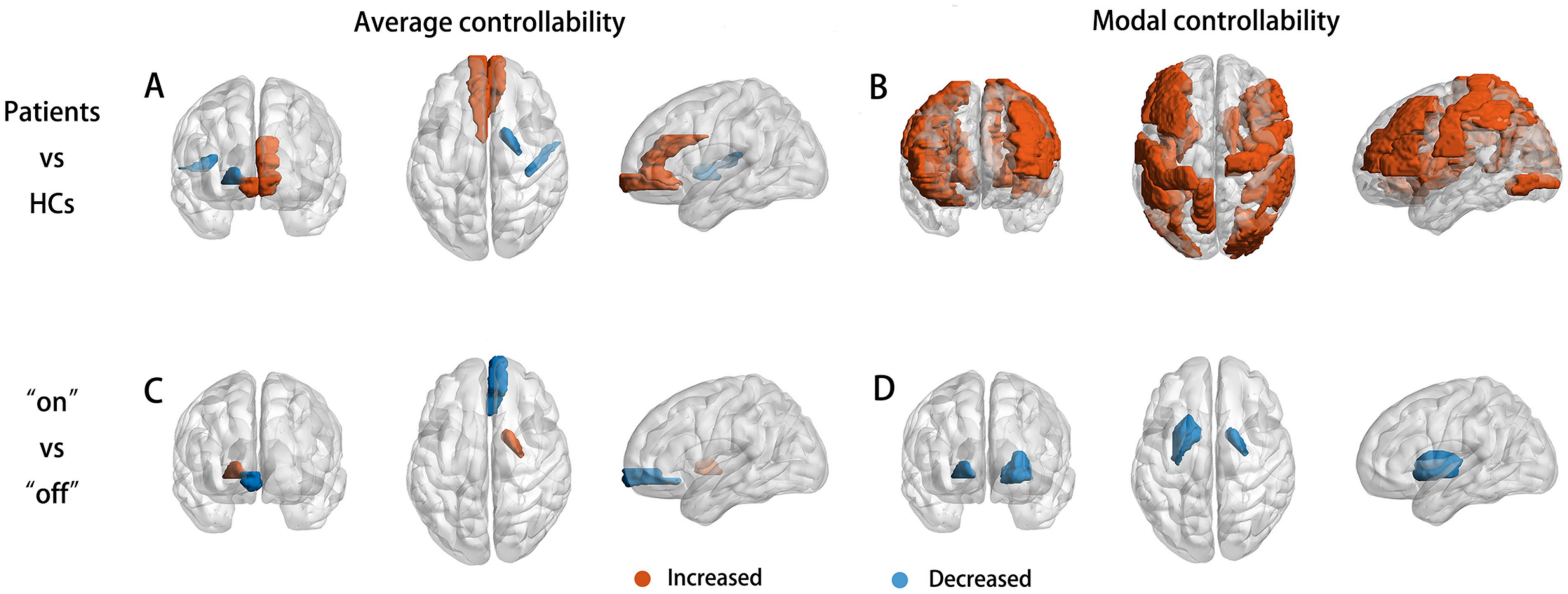
The brain regions with aberrant controllability responsive to medication. Compared to HCs, significant increase or decrease were found in the AC **(A)** and MC **(B)** of each brain node of patients with PD. After medication, aberrant AC of the right pallidum significantly increased and which of the right orbital middle frontal gyrus significantly decreased **(C)**. Aberrant MC of the bilateral pallidum and the left putamen significantly decreased **(D)**, displaying a trend of normalization of network controllability in the striatal areas introduced by medication.

Compared to the HCs, patients with PD at preoperative “off” state showed increased AC in the middle frontal gyrus and cingulum gyrus, decreased AC in the globus pallidum and Heschl’s gyrus, and increased MC in the precentral gurus, middle and inferior frontal gyrus, insula gyrus, superior and middle and inferior occipital gyrus, postcentral gyrus, inferior parietal gyrus, supramarginal gyrus, precuneus, caudate nucleus, putamen, globus pallidum, and Heschl’s gyrus (Figure 1; Table 3). Between preoperative medication “on” and “off” states, the AC in the right orbital middle frontal gyrus, MC in left putamen and bilateral pallidum were significantly decreased, and the AC in the right pallidum was significantly increased, displaying a trend of normalization of network controllability for patients after medication.

**Table 3:** Aberrant brain regions of controllability responsive to medication.

Controllability	Brain region^a^	Hemisphere	Patients and HCs			After and before medication^c^	
			F	*p*	Changes^b^	T	*p*
Average	ORBmid	Left	4.860	0.031	+	NS	NS
Average	ORBmid	Right	6.778	0.012	+	−2.984	0.006
Average	ACG	Left	13.123	0.001	+	NS	NS
Average	PAL	Right	4.406	0.040	−	2.280	0.030
Average	HES	Right	4.267	0.043	−	NS	NS
Modal	PreCG	Right	4.123	0.047	+	NS	NS
Modal	MFG	Left	4.091	0.047	+	NS	NS
Modal	IFGtriang	Left	6.124	0.016	+	NS	NS
Modal	IFGtriang	Right	4.507	0.038	+	NS	NS
Modal	ORBinf	Right	4.221	0.044	+	NS	NS
Modal	INS	Left	4.048	0.049	+	NS	NS
Modal	INS	Right	4.505	0.038	+	NS	NS
Modal	SOG	Right	4.000	0.050	+	NS	NS
Modal	MOG	Right	5.785	0.019	+	NS	NS
Modal	IOG	Left	4.833	0.032	+	NS	NS
Modal	PoCG	Left	4.878	0.031	+	NS	NS
Modal	IPL	Left	7.705	0.007	+	NS	NS
Modal	IPL	Right	5.855	0.018	+	NS	NS
Modal	SMG	Right	6.214	0.015	+	NS	NS
Modal	PCUN	Left	4.782	0.033	+	NS	NS
Modal	CAU	Right	4.345	0.041	+	NS	NS
Modal	PUT	Left	7.130	0.010	+	−2.101	0.044
Modal	PAL	Left	9.953	0.002	+	−2.950	0.006
Modal	PAL	Right	8.382	0.005	+	−2.497	0.018
Modal	HES	Left	6.166	0.016	+	NS	NS
Modal	HES	Right	7.257	0.009	+	NS	NS

a ORBmid, Middle frontal gyrus, orbital part; ACG, Anterior cingulate and paracingulate gyri; PAL, Lenticular nucleus, pallidum; HES, Heschl gyrus; PreCG, Precentral gyrus; MFG, Middle frontal gyrus; IFGtriang, Inferior frontal gyrus, triangular part; ORBinf, Inferior frontal gyrus, orbital part; INS, Insula lobe; SOG, Superior occipital gyrus; MOG, Middle occipital gyrus; IOG, Inferior occipital gyrus; PoCG, Postcentral gyrus; IPL, Inferior parietal lobe; SMG, Supramarginal gyrus; PCUN, Precuneus lobe; CAU, Caudate nucleus; PUT, Lenticular nucleus, putamen.

b Increase (+) or decrease (−) changes were found in the controllability of the patients compared to HCs.

c NS, No significant difference was found in the controllability between the medication “on” and “off” states.

### 3.3 Correlation between delta controllability and the improvement rate of UPDRS-III

The association between changes in the controllability of focal regions after medication and the motor outcomes of STN-and GPi-DBS in patients with PD was evaluated. In Table 1, in the STN group, the improvement rates of UPDRS-III after DBS were negatively correlated with delta AC of bilateral thalamus. In the GPi group, the improvement rates of UPDRS-III were positively correlated with the delta AC of the bilateral postcentral gyrus, negatively correlated with the delta MC of the left postcentral gyrus, and positively correlated with the delta MC of the left caudate nucleus.

### 3.4 Prediction of improvement rate of UPDRS-III in the STN and GPi groups

**Figure 2:**
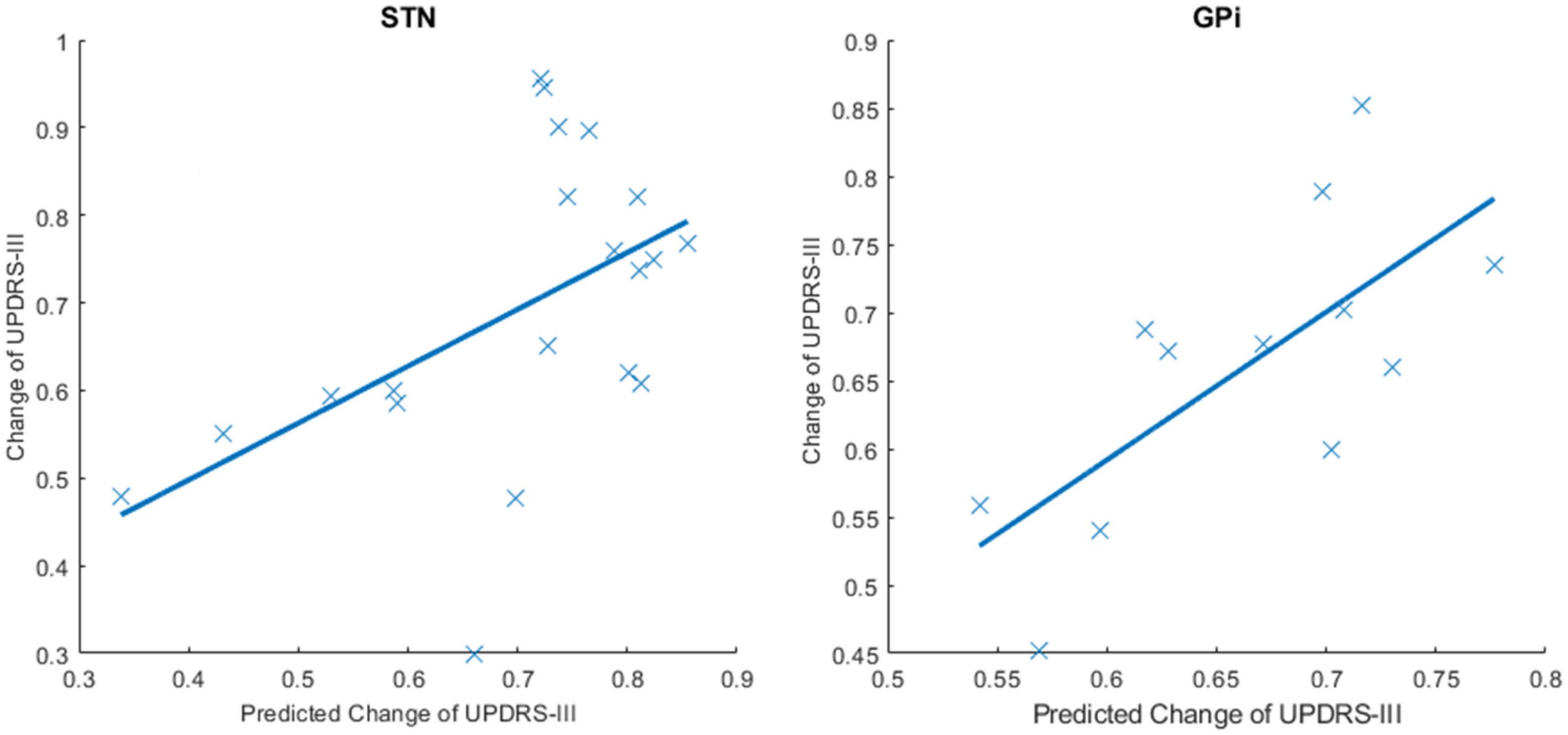
Predicted and actual changes of the UPDRS-III scores in the STN and GPi groups. SVR models were conducted to predict the improvement rates of the UPDRS-III scores using delta controllability. This figure shows the predicted and actual changes in the UPDRS-III scores after DBS surgery. The predicted changes in the UPDRS-III scores and the actual changes in the UPDRS-III scores were positively correlated in the STN group (*p* = 0.020 and *R* = 0.514) and the GPi group (*p* = 0.011 and *R* = 0.705).

As shown in Figure 2, in the STN group, changes in the average controllability of both sides of the thalamus were selected as predictive features. The predicted and actual changes in the UPDRS-III scores were positively correlated (*p* = 0.020 and *R* = 0.514, respectively). In the GPi group, the delta AC of the postcentral gyrus, delta MC of the left postcentral gyrus, and left caudate nucleus were selected for prediction. The predicted and actual changes in the UPDRS-III scores were also positively correlated (*p* = 0.011 and *R* = 0.705, respectively).

## 4 Discussion

The current study revealed different circuits involved in STN-DBS and GPi-DBS by studying baseline and change of network controllability between medication “on” and “off” states, and made the first attempt to predict the motor outcomes of PD patients after DBS using preoperative functional controllability. Our results confirm the significance of striatal areas in network dysfunction and demonstrate the normalization effect of levodopa on these dysfunctional regions in PD. Moreover, we found that the motor outcomes of DBS were associated with preoperative controllability of different nodes between the STN-DBS and GPi-DBS groups and highlighted the predictive value of functional controllability of the thalamus in STN-DBS and caudate nucleus and postcentral gyrus in GPi-DBS.

Compared to HCs, patients with PD showed a decreased AC and increased MC in wide areas of motor-related regions of the brain. In striatal areas, decreased AC indicated a lower network degree or node strength. Similarly, increased MC suggested sparser connections with other brain areas (Gu et al., 2015), consistent with previous studies that found significantly reduced functional connectivity between the striatal nuclei and cortical areas in patients with PD (Baggio et al., 2015; Ruppert et al., 2020; Hacker et al., 2012; Herz et al., 2021). However, several studies reported the vital role of global integration of dynamic functional connectivity networks instead of specific abnormal connections in PD (Tessitore et al., 2019; Kim et al., 2017). AC is defined as the diagnostic evaluating a node’s ability to drive the network system into easy-to-reach states, while MC identifies brain areas that can steer the system into difficult-to-reach states (Gu et al., 2015; Li et al., 2023). In this context, regions of the striatum show a decreased ability to guide the transition of brain networks into desirable network states in patients with PD. Striatal areas and widespread motor-related cortices of the frontal, parietal, and occipital lobes with an increase in MC indicate a tendency for these regions to drive the brain system into difficult-to-reach states, with higher energy costs for task completion. Increased MC suggests that these regions are in difficult-to-reach states, with higher energy costs for task completion. In general, as reported in a previous study (Kim et al., 2017), a more frequent and sparsely connected state decreased, and a less frequent and stronger interconnected state increased in PD. In contrast, increased AC in the frontal lobe and cingulate gyrus indicates an increased compensatory ability in some hubs to drive brain networks into desirable states.

Between the medication “on” and “off” states, significant change of controllability metrics was found in striatal regions including putamen and globus pallidum, and orbital part of middle frontal gyrus, suggesting a reversion of abnormal network controllability in these regions. Striatal dysfunction has been reported as a core abnormality in PD and can be reversed by restorative dopaminergic effect (Xing et al., 2020). Dopaminergic medications do not simply normalize PD-related connectivity changes but uniquely alter connectivity with the striatum and medial prefrontal cortex (Ng et al., 2017). Increased AC and decreased MC in the striatal areas indicate an improvement in steering the brain system into easy-to-reach states with lower energy costs, which is consistent with clinical improvement after medication. Our results suggest that striatal areas play an important role in network dysfunction in PD and are an important target region for dopaminergic drugs in PD.

The STN and GPi are the two most common stimulation sites within the CSTC motor loops (Zhang et al., 2021; Chu, 2022). Our results suggest that different nodes within the CSTC motor loop contribute differently to motor improvements following STN or GPi stimulation. In the STN-DBS group, the improvement rates of UPDRS-III were negatively related to delta AC in the thalamus, indicating that a greater increment in AC or better medication response of the thalamus on controllability might lead to better motor outcomes after DBS. The thalamus and the subthalamic nucleus are structurally and functionally connected, sharing essential projections which contribute to movement initiation in PD (Watson et al., 2021; Chen et al., 2023). As an essential region for motor modulation in STN-DBS, the thalamus exhibits dynamic changes in its functional networks during stimulation. Stimulation with STN-DBS has been reported to increase connectivity between the motor cortex and thalamus (Mueller et al., 2018) and alter corticothalamic coupling in patients with PD (Zhang et al., 2021; Kahan et al., 2012). The thalamus is also a core hub of the cerebellothalamocortical (CTC) circuit, which is associated with the pathology underlying tremor-dominant (TD) subtypes of PD (Zhang et al., 2015; Zhong et al., 2023). As an appropriate treatment for TD patients, STN-DBS can improve motor outcomes by affecting the CTC circuit (Luo, 2021). Medication response to motor symptoms has been shown to be related to DBS motor outcomes (Machado et al., 2006; Lin et al., 2021; Zheng, 2021); however, few studies have reported a relationship between medication response to brain dynamics and DBS motor outcomes. In the current study, although significant differences in controllability were not found in the thalamus after medication, the medication response reflected by delta AC in the thalamus was related to DBS outcomes in the STN-DBS group. If the thalamus has a greater ability to drive the brain into easy-to-reach states introduced by medication, then better motor performance can be observed in patients with PD after STN-DBS.

In the GPi-DBS group, the improvement rates of UPDRS-III were positively associated with the delta AC of the postcentral gyrus, negatively associated with the delta MC of the postcentral gyrus, and positively associated with the delta MC of caudate nucleus. The striatum has been reported to be an important hub for motor modulation in PD (Hou et al., 2016), as confirmed in the current study. The caudate nucleus, an important part of the striatum, was found to decrease information transfer introduced by GPi-DBS (Chu, 2022). After medication, a greater decrease in MC or a better medication response of the caudate nucleus might lead to better motor outcomes with GPi-DBS. This is in accordance with the knowledge that dopaminergic drugs act by modulating the striatal regions. The postcentral gyrus, which is a part of the sensorimotor cortex, shows abnormal connectivity with other motor-related cortices in the early stages of PD (Tuovinen et al., 2018). Increased connectivity was found in the caudate in patients with PD, whereas decreased connectivity was found in the postcentral gyrus (Wang et al., 2023). In the current study, an abnormal MC was found in the postcentral gyrus; however, no significant difference was observed after medication. Less decrement of MC and less increment of AC, or less medication effect on the postcentral gyrus, might lead to lower post-surgery UPDRS-III scores or better motor outcomes of GPi-DBS. An intense response to medication is not always related to satisfactory DBS motor outcomes in different brain regions. A greater response in the striatum and more stable sensorimotor areas under medication effects may lead to better motor outcomes in patients undergoing GPi-DBS.

We further validated the predictive value of delta controllability of focal nodes for DBS motor outcomes. In the SVR model, the medication response of the thalamus, revealed by delta controllability, showed good performance in predicting the motor outcomes of STN-DBS, and the medication response of the caudate and postcentral gyrus could predict the motor outcomes of GPi-DBS. Our results are consistent with those of previous studies and highlight the important role of the thalamus in the motor modulation of STN-DBS, and of the caudate nucleus and postcentral gyrus in the motor modulation of GPi-DBS, illustrating the promise of network controllability of the thalamus, striatal regions, and sensorimotor cortex in predicting motor outcomes of DBS.

Our study has several limitations. First, only short-term motor outcomes were examined. For patients with PD, long-term motor outcomes may have a greater impact on their quality of life, which will be explored in the future. Second, in addition to motor symptoms, nonmotor symptoms are important indicators of DBS outcomes. Although we found no difference in non-motor symptoms before surgery between the STN-and GPi-DBS groups, postoperative changes, especially the potential negative effects of DBS on non-motor symptoms, should be considered in the future. Finally, the network control theory reduces the entire brain to a linear system. Although many studies have demonstrated the feasibility of linear systems and the reliability of network control theory, when facing the complex systems of the brain, we still need to find more evidence and continue exploring.

In summary, we confirmed the key role of the striatum in network dysfunction and levodopa response in PD and the predictive value of functional controllability of the thalamus in STN-DBS and striatal areas in GPi-DBS. Our findings indicate that different focal regions within the CSTC motor loops are involved in STN-DBS and GPi-DBS, and support the feasibility of brain-controllability-based biomarkers to mechanistically understand and predict the motor effects of DBS for Parkinson’s disease in clinical decision-making.

## Data Availability

The original contributions presented in the study are included in the article/Supplementary material, further inquiries can be directed to the corresponding authors.

## Footnotes

^1^ http://www.fil.ion.ucl.ac.uk/spm/

^2^ https://complexsystemsupenn.com/codedata
