# Supplementary material for: Functional brain controllability in Parkinson’s disease and its association with motor outcomes after deep brain stimulation
Source: Front Neurosci. 2024 Nov 7;18:1433577. doi: 10.3389/fnins.2024.1433577 (PMC11578951; doi:10.3389/fnins.2024.1433577)
Supplement: Supplementary file 1 [file Data_Sheet_1.docx]

Supplementary Material

# Supplementary Data

## Prediction of Improvement rate of UPDRS-III in all patients using delta controllability

We constructed SVR models based on the features selected in SVR models of STN-DBS and GPi-DBS subgroups (delta AC of both sides of thalamus and the postcentral gyrus, delta MC of the left postcentral gyrus and left caudate nucleus) to predict the improvement rates of UPDRS-III after DBS surgery across all patients. This model showed suboptimal performance, with no significant correlation between the predicted and actual values (p = 0.837 and r = 0.038).

## The predictive power of levodopa responsiveness

The percentage change in the UPDRS-III score between the preoperative medication-on and medication-off states was calculated, termed as levodopa responsiveness (LR). Pearson correlation analysis showed no significant correlation between LR and postoperative improvement rate of UPDRS-III (p = 0.347, r = 0.201 in the STN-DBS group, and p = 0.636, r = 0.128 in Gpi-DBS group). The SVR model based on LR also did poor predictive performance, with no significant correlations in the predicted and actual postoperative improvement rate (p <0.001, r = -0.742 in the STN-DBS group, and p = 0.202, r = -0.337 in the GPi-DBS group).

## Relationship between levodopa responsiveness and the normalization of controllability

In this study, we believe that the controllability of the right orbital middle frontal gyrus, left putamen and bilateral pallidum show a trend toward normalization under the influence of medication. However, preoperative LR was not significantly correlated with the delta controllability of these brain regions (Table S1). Subjects with good levodopa response weren’t observed to have more normalization of controllability in our study.

## Results with head motion scrubbing

Resting-state functional MRI scans were preprocessed using Statistical Parametric Mapping (SPM12, http://www.fil.ion.ucl.ac.uk/spm/) and Data Processing & Analysis Brain Imaging (DPABI, version 6.0)(1) based on the MATLAB 2017b platform with the pipeline including format conversion, removing the first 12 time points, slice timing correction, head motion correction, spatial normalization to the Montreal Neurological Institute (MNI) template and smooth. Then, the global mean signal, 24 head motion parameters, white matter signal, and cerebrospinal fluid signal were regressed to reduce the effect of head motion on functional connectivity. Next, a band-pass filter (0.01–0.08 Hz) was used to remove low- or high- frequency noise and artifacts. We checked the images and head motion parameters after normalization and removed scans with head motion of more than 3 mm. Finally, we scrubbed the time points with framewise displacement (FD) > 0.5 mm, as well as volumes 2 forward and 1 back from these volumes as recommended(1, 2). Functional connectivity (FC) metrics were constructed from 90 regions of interest (ROIs) parcellated using an automated anatomical labeling (AAL) atlas. Then, we calculated the AC and MC from functional connectivity of each HC and each patient at both medication “on” and “off” states.

**1.4.1 Aberrant Brain Regions of Controllability Responsive to Medication**

We conducted covariance analyses to compare differences in AC and MC between patients with PD and HCs, and paired t-tests to measure the changes in the AC and MC before and after medication in patients with PD. We counted brain regions that not only had significant differences between patients and HCs but also changed significantly after medication.

Similar to the original results, the AC in the right orbital middle frontal gyrus, MC in left putamen and bilateral pallidum were significantly decreased, displaying a trend of normalization of network controllability for patients after medication. A similar trend was also found in the AC of the right pallidum although no significant differences was found between patients and HCs in this region. (Table S2).

**1.4.2 Correlation Between Delta Controllability and the Improvement Rate of UPDRS-III**

Pearson’s correlations between delta controllability of the basal ganglia and cortical sensorimotor areas (caudate nucleus, putamen, globus pallidum, thalamus, precentral gyrus, postcentral gyrus, supplementary motor area, and paracentral lobule) and improvement rates of UPDRS-III after DBS surgery were conducted in the STN and GPi groups.

Similar to the original results, in the STN group, the improvement rates of UPDRS-III after DBS were negatively correlated with delta AC of bilateral thalamus. In the GPi group, the improvement rates of UPDRS-III were positively correlated with the delta AC of the bilateral postcentral gyrus, and positively correlated with the delta MC of the left caudate nucleus. There was also a similar trend for the delta MC of the left postcentral gyrus in the GPi group compared to the original results (Table S3).

**1.4.3 Prediction of Improvement rate of UPDRS-III in the STN and GPi Groups**

SVR models were conducted to explore whether delta controllability in the regions found in Table S3 could predict the improvement rates of UPDRS-III after DBS surgery in the STN-DBS and GPi-DBS groups. In the STN group, the delta AC of bilateral thalamus were selected as predictive features. In the GPi group, the delta AC of the postcentral gyrus and the delta MC of the left caudate nucleus were selected for prediction.

The predicted and actual changes in the UPDRS-III scores were also positively correlated in the STN group (P = 0.035 and R = 0.475) and in the GPi group (P = 0.006 and R = 0.732) (Figure S1).

# Supplementary Tables

**Table S1 Correlation between Levodopa responsiveness and normalization of controllability**

| **Controllability** | **Brain region** | **Hemisphere** | **Patients vs HCs** | | | **After vs before medication** | | **Correlation between LR and delta controllability** | |
| --- | --- | --- | --- | --- | --- | --- | --- | --- | --- |
|  |  |  | **F** | **p** | **Changes** | **T** | **p** | **R** | **p** |
| Average | ORBmid^a^ | Right | 6.778 | 0.012 | + | -2.984 | 0.006 | 0.207 | 0.255 |
| Average | PAL^b^ | Right | 4.406 | 0.040 | - | 2.280 | 0.030 | 0.082 | 0.657 |
| Modal | PUT^c^ | Left | 7.130 | 0.010 | + | -2.101 | 0.044 | 0.050 | 0.787 |
| Modal | PAL | Left | 9.953 | 0.002 | + | -2.950 | 0.006 | 0.046 | 0.803 |
| Modal | PAL | Right | 8.382 | 0.005 | + | -2.497 | 0.018 | 0.082 | 0.655 |

^a^ ORBmid = Middle frontal gyrus, orbital part;

^b^ PAL = Lenticular nucleus, pallidum;

^c^ PUT = Lenticular nucleus, putamen.

**Table S2 Aberrant Brain Regions of Controllability Responsive to Medication**

| **Controllability** | **Brain region**^a^ | **Hemisphere** | **Results without motion scrubbing** | | | | | **Results with motion scrubbing** | | | | |
| --- | --- | --- | --- | --- | --- | --- | --- | --- | --- | --- | --- | --- |
|  |  |  | **Patients and HCs** | | | **After and before medication** | | **Patients and HCs** | | | **After and before medication** | |
|  |  |  | **F** | **p** | **Changes**^b^ | **T** | **p** | **F** | **p** | **Changes** | **T** | **p** |
| Average | ORBmid | Left | 4.860 | 0.031 | + | NS^c^ | NS | 5.325 | 0.024 | + | NS | NS |
| Average | ORBmid | Right | 6.778 | 0.012 | + | -2.984 | 0.006 | 7.953 | 0.006 | + | -2.722 | 0.011 |
| Average | ACG | Left | 13.123 | 0.001 | + | NS | NS | 14.840 | 0.000 | + | NS | NS |
| Average | PAL | Right | 4.406 | 0.040 | - | 2.280 | 0.030 | NS | NS | / | 2.312 | 0.028 |
| Average | HES | Right | 4.267 | 0.043 | - | NS | NS | 8.905 | 0.004 | - | NS | NS |
| Modal | PreCG | Right | 4.123 | 0.047 | + | NS | NS | 6.102 | 0.016 | + | NS | NS |
| Modal | MFG | Left | 4.091 | 0.047 | + | NS | NS | 5.818 | 0.019 | + | NS | NS |
| Modal | IFGtriang | Left | 6.124 | 0.016 | + | NS | NS | 6.902 | 0.011 | + | NS | NS |
| Modal | IFGtriang | Right | 4.507 | 0.038 | + | NS | NS | 4.037 | 0.049 | + | NS | NS |
| Modal | ORBinf | Right | 4.221 | 0.044 | + | NS | NS | NS | NS | / | NS | NS |
| Modal | INS | Left | 4.048 | 0.049 | + | NS | NS | 4.964 | 0.030 | + | NS | NS |
| Modal | INS | Right | 4.505 | 0.038 | + | NS | NS | 6.231 | 0.015 | + | NS | NS |
| Modal | SOG | Right | 4.000 | 0.050 | + | NS | NS | NS | NS | / | NS | NS |
| Modal | MOG | Right | 5.785 | 0.019 | + | NS | NS | 4.820 | 0.032 | + | NS | NS |
| Modal | IOG | Left | 4.833 | 0.032 | + | NS | NS | 5.157 | 0.027 | + | NS | NS |
| Modal | PoCG | Left | 4.878 | 0.031 | + | NS | NS | 4.411 | 0.040 | + | NS | NS |
| Modal | IPL | Left | 7.705 | 0.007 | + | NS | NS | 8.612 | 0.005 | + | NS | NS |
| Modal | IPL | Right | 5.855 | 0.018 | + | NS | NS | 7.591 | 0.008 | + | NS | NS |
| Modal | SMG | Right | 6.214 | 0.015 | + | NS | NS | 8.210 | 0.006 | + | NS | NS |
| Modal | PCUN | Left | 4.782 | 0.033 | + | NS | NS | 7.387 | 0.009 | + | NS | NS |
| Modal | CAU | Right | 4.345 | 0.041 | + | NS | NS | 4.236 | 0.044 | + | NS | NS |
| Modal | PUT | Left | 7.130 | 0.010 | + | -2.101 | 0.044 | 8.440 | 0.005 | + | -2.313 | 0.028 |
| Modal | PAL | Left | 9.953 | 0.002 | + | -2.950 | 0.006 | 11.869 | 0.001 | + | -3.168 | 0.003 |
| Modal | PAL | Right | 8.382 | 0.005 | + | -2.497 | 0.018 | 10.558 | 0.002 | + | -2.702 | 0.011 |
| Modal | HES | Left | 6.166 | 0.016 | + | NS | NS | 7.718 | 0.007 | + | NS | NS |
| Modal | HES | Right | 7.257 | 0.009 | + | NS | NS | 10.990 | 0.002 | + | NS | NS |

^a^ ORBmid = Middle frontal gyrus, orbital part; ACG = Anterior cingulate and paracingulate gyri; PAL = Lenticular nucleus, pallidum; HES = Heschl gyrus; PreCG = Precentral gyrus; MFG = Middle frontal gyrus; IFGtriang = Inferior frontal gyrus, triangular part; ORBinf = Inferior frontal gyrus, orbital part; INS = Insula lobe; SOG = Superior occipital gyrus; MOG = Middle occipital gyrus; IOG = Inferior occipital gyrus; PoCG = Postcentral gyrus; IPL = Inferior parietal lobe; SMG = Supramarginal gyrus; PCUN = Precuneus lobe; CAU = Caudate nucleus; PUT = Lenticular nucleus, putamen.

^b^ Increase (+) or decrease (-) changes were found in the controllability of the patients compared to HCs.

^c^ NS: No significant difference was found.

**Table S3 Correlation between delta controllability and improvement rates of the UPDRS-III scores**

| **Location** | **Type of Controllability** | **brain region**^a^ | **Hemisphere** | **Results without motion scrubbing** | | **Results with motion scrubbing** | |
| --- | --- | --- | --- | --- | --- | --- | --- |
|  |  |  |  | **R** | **p** | **R** | **p** |
| STN | Average | THA | Left | -0.687 | 0.001 | -0.601 | 0.005 |
| STN | Average | THA | Right | -0.624 | 0.003 | -0.531 | 0.016 |
| GPi | Average | PoCG | Left | 0.744 | 0.006 | 0.700 | 0.011 |
| GPi | Average | PoCG | Right | 0.643 | 0.024 | 0.619 | 0.032 |
| GPi | Modal | PoCG | Left | -0.584 | 0.046 | -0.564 | 0.056 |
| GPi | Modal | CAU | Left | 0.592 | 0.042 | 0.621 | 0.031 |

^a^ THA = Thalamus; PoCG = Postcentral gyrus; CAU = Caudate nucleus.

**3 Supplementary Figure**

**
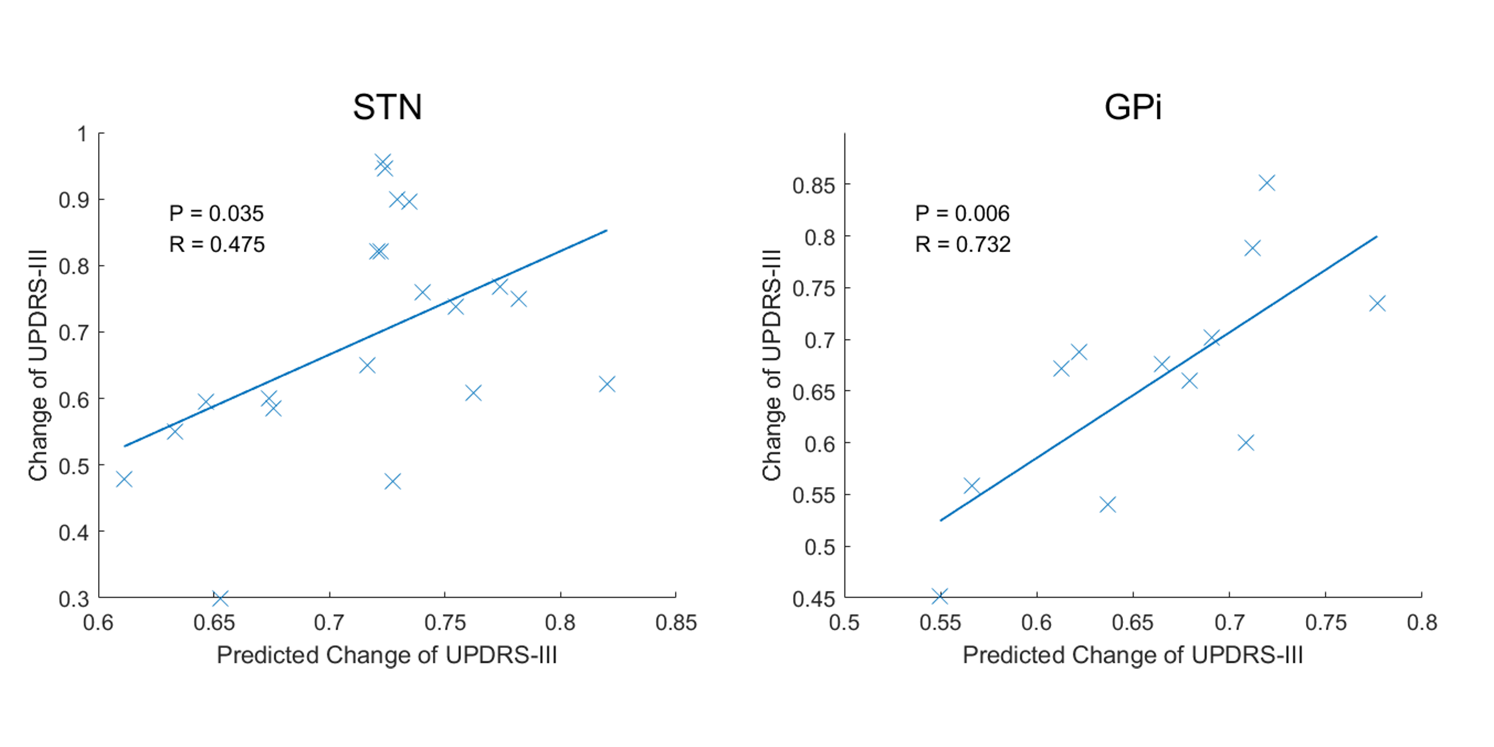
**

**Fig.S1 Predicted and actual changes of the UPDRS-III scores in the STN and GPi groups**

This figure shows the predicted and actual changes in the UPDRS-III scores after DBS surgery. The predicted changes in the UPDRS-III scores and the actual changes in the UPDRS-III scores were positively correlated in the STN group (P = 0.035 and R = 0.475) and in the GPi group (P = 0.006 and R = 0.732).

# 4 Reference

1. Yan, C.G., et al., *DPABI: Data Processing & Analysis for (Resting-State) Brain Imaging.* Neuroinformatics, 2016. **14**(3): p. 339-51.

2. Jenkinson, M.,et al., *Improved optimization for the robust and accurate linear registration and motion correction of brain images.* NeuroImage, 2002.17(2): P. 825–841.
